# Recent Advances in Novel Packaging Technologies for Shelf-Life Extension of Guava Fruits for Retaining Health Benefits for Longer Duration

**DOI:** 10.3390/plants11040547

**Published:** 2022-02-18

**Authors:** Ajay Yadav, Nishant Kumar, Ashutosh Upadhyay, Olaniyi Amos Fawole, Manoj Kumar Mahawar, Kirti Jalgaonkar, Deepak Chandran, Sureshkumar Rajalingam, Gokhan Zengin, Manoj Kumar, Mohamed Mekhemar

**Affiliations:** 1 Agro Produce Processing Division, ICAR—Central Institute of Agricultural Engineering, Bhopal 462038, India; ajay.yadav1@icar.gov.in; 2 Department of Food Science and Technology, National Institute of Food Technology Entrepreneurship and Management, Sonepat 131028, India; nishant.kumar@niftem.ac.in; 3 Postharvest Research Laboratory, Department of Botany and Plant Biotechnology, University of Johannesburg, Auckland Park, Johannesburg P.O. Box 524, South Africa; olaniyif@uj.ac.za; 4 Technology Transfer Division, ICAR—Central Institute for Research on Cotton Technology, Mumbai 400019, India; manojmahawar362@gmail.com; 5 Quality Evaluation and Improvement Division, ICAR—Central Institute for Research on Cotton Technology, Mumbai 400019, India; jalgaonkar.kirti@gmail.com; 6 Department of Veterinary Sciences and Animal Husbandry, Amrita School of Agricultural Sciences, Amrita Vishwa Vidyapeetham University, Coimbatore 642109, India; c_deepak@cb.amrita.edu; 7 Department of Agronomy, Amrita School of Agricultural Sciences, Amrita Vishwa Vidyapeetham University, Coimbatore 642109, India; r_sureshkumar@cb.amrita.edu; 8 Physiology and Biochemistry Research Laboratory, Department of Biology, Science Faculty, Selcuk University, Konya 42130, Turkey; gokhanzengin@selcuk.edu.tr; 9 Chemical and Biochemical Processing Division, ICAR—Central Institute for Research on Cotton Technology, Mumbai 400019, India; 10 Clinic for Conservative Dentistry and Periodontology, School of Dental Medicine, Christian-Albrecht’s University, 24105 Kiel, Germany

**Keywords:** physiological disorder, oxidation, packaging technologies, MAP and CAP, edible packaging, nano and smart packaging, shelf-life extension

## Abstract

Guava (*Psidium guajava* L.) fruit is also known as the apple of tropics, belongs to the family of genus Psidium, and is widely cultivated in tropical zones of the world. Recently, the importance of guava fruit has increased due to its inherent nutritional content, pleasant aroma, excellent flavor, and delicious taste. It is considered an excellent source of nutrients and phytochemicals. Guava is a climacteric fruit that continues to mature or ripen even after harvest, showing an increase in the rate of respiration and metabolic activities within a short period, leading to rapid senescence or spoilage of fruit. It has limitations in terms of commercialization due to short storage life after harvest and sensitivity to diseases and chilling injury during the storage period. Many postharvest technologies such as edible packaging, modified atmosphere packaging (MAP), composite packaging, controlled atmosphere packaging (CAP), antimicrobial/antifungal packaging, and nano packaging have been used to retard the chilling injury and enhance the keeping quality of guava fruits during the storage period to control respiration rate, reduce weight loss, minimize lipid oxidation, and maintain organoleptic properties. However, these packaging technologies have varied effects on the internal and external quality attributes of guava fruits. This review, therefore, discusses the physiology, mechanism of ripening, oxidation, and ethylene production of guava fruits. The review also discusses the packaging technologies and their effect on the postharvest characteristics of guava fruits during the storage period.

## 1. Introduction

Guava (*Psidium guajava* L.) is considered one of the most vital fruits, is also known as the apple of tropical and subtropical countries, and has experienced high consumer demand [1,2,3,4]. In the last decade, the demand for minimally processed agriculture and horticultural products with freshness, nutritional quality, and safety has increased worldwide among consumers [5]. The commercial demand for guava fruits is increasing due to their inherent nutritional content, pleasant aroma, excellent flavor, and delicious taste. It contains a good amount of ascorbic acid; pectin; dietary fibers; and minerals such as iron, calcium, and phosphorus. The presence of phytochemicals in guava has serum-cholesterol-lowering properties and hepato-protective effects on human health [6,7,8,9]. Guava is generally consumed fresh, or converted into different value-added products such as jam and jellies, and production of guava is estimated to be 4.05 million metric tons in 2017–2018 [10]. The most popular and commercial varieties of guava fruits are Allahabad Safed, Luckone-49, Nagpur Seedless, Dharwar, Baraipur, etc. [3,11]. Guava is a climacteric fruit that continues to mature or ripen even after harvest, showing an increase in the rate of respiration and metabolic activities within a short period of time, leading to rapid senescence or spoilage of fruit. Due to its limited postharvest shelf life and sensitivity to diseases and chilling injuries during storage, it poses constraints for commercialization. Usually, it has short postharvest shelf life of 3–4 days at 25 ± 2 °C and 2–3 weeks at 7–10 °C, while storing it below 13 °C results in chilling injuries [11,12,13,14,15,16,17,18].

The post-harvest losses of guava in developing countries are to tune of 20–40% of the produce. In countries such as India, among fruits, the maximum post-harvest losses occur in guava fruit (nearly 18.1%), including 4.1% storage losses and 3.7% packaging and transportation losses [19]. Due to lack of packaging facilities and the improper storage of guava, there is a huge loss in physiological weight, changes in total soluble solid (TSS), and vitamin C, and guava-browning has been widely documented in the literature. Many researchers have concluded that the rate of oxygen consumption and the evolution of CO_2_ and ethylene production during the packaging and storage of fruit play a major role in extending shelf life [3,7,20,21,22,23,24,25]. Modified atmospheric packaging (MAP), controlled atmospheric packaging (CAP), and edible packaging have significantly controlled the deterioration of fruit for a longer period of time by maintaining the proper gaseous environment around the fruit [18,26,27,28]. These technologies are aimed at reducing the respiration rate and transpiration rate and subsequently degrade the speed of biochemical reaction occurring during the storage of fruit. MAP and CAP technologies make use of polymeric films such as LDPE and PP, for the packaging of guava. These films are made up of plastic and possess a threat to the environment and human beings [24,29,30,31]. So, to minimize the risk associated with plastic-based film, the use of biodegradable films and edible coatings has become popular in recent times. Edible films and coatings are the new trends for the coating of guava, to extend its shelf life [32,33,34,35,36,37,38]. The relevant studies on different types of novel postharvest management strategies and packaging technologies for the shelf-life extension of guava fruits have included searches using different data bases (Scopus, Web of science, PubMed, Google Scholar, Research Gate, etc.). In this regard, more than 300 research articles, review articles, and book chapters were identified, and 180 studies were found relevant and cited as references in the current review. There is no dedicated review available on recent advances in packaging that exclusively talks about the packaging of guava with an emphasis on recent developments in the edible coating of guava. Keeping all these in view, this review aims at reviewing (i) the mechanism associated with the respiration rate and transpiration rate of guava; and (ii) recent developments in packaging technologies (MAP, CAP, edible films or coatings) and their influence on the post-harvest management of guava fruits.

## 2. Physiology of Guava Fruits

In the guava plant, flowering and fruiting occur throughout the year under tropical and subtropical climates. The growth curve of guava fruit is depicted as double sigmoidal growth and can be divided into three distinctive stages: (i) fast phase of growth that takes place just after anthraces and generally for 45–60 days; (ii) slow growth rate phase, which is generally for 30–60 days and characterized by hardening and maturation of seeds; (iii) exponential phase, in which fruit attain maturity and time period is of 30–60 days [3,39,40]. Maturity is a stage of fruit development at which quality attributes such as firmness, nutrition value, and appearance (color, shape, and size) are acceptable to the consumers. The maturity of guava fruit depends upon the number of days from full bloom to the day till it reaches the harvest maturity, which varies from variety to variety and environmental conditions, for instance, it is generally 120–150 days during the spring season and 90–105 days for the winter season [3,24]. For proper storage and packaging of guava, the fruit should be plucked at optimum harvesting maturity, which is determined by measuring the maturity indices. Maturity indices such as specific gravity, visual appearance, firmness, and chemical composition are indicators for the development of the marketable quality of guava during the ripening process. Guava is having four maturity indices such as mature green, turning green, ripe, and overripe [11,24,41,42].

### 2.1. Mechanism of Ripening

**Figure 1:**
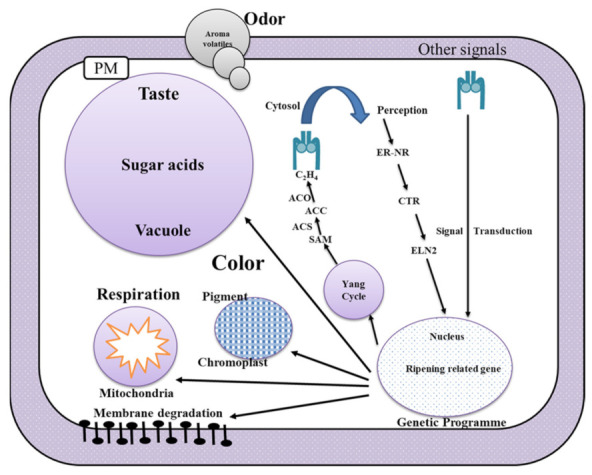
Mechanism of fruit ripening (modified from Bouzayen et al. [44]).

Ripening of guava is a series of physiological, biochemical, structural changes that occurs in unripe fruit, which makes it more palatable with good quality attributes such as shifting of skin color from green to yellowish tint, softening of texture, development of characteristic aroma, and flavor in the fruit. Figure 1 shows the mechanism of fruit ripening during the storage period. The change in skin color during guava ripening is attributed to the presence of chlorophyll and carotenoids pigments, which vary from variety to variety. The production of ethylene in fruits induced the loss of chlorophyll, anthocyanin, and other activation of enzymes, i.e., PPO/POD [43]. Bouzayen et al. [44] reported the ripening of the fruits as a result, in which physiology and biochemistry of the organs are developed to influence the properties of fruits such as texture, flavor, color, aroma, and appearance. Jain et al. [3] reported the total carotenoid content in guava cv. *Banarsi Surkhai* was increased from 0.34 to 0.45 (mg/100 cm^2^), whereas chlorophyll content decreased from 1.24 to 1.01 (mg/100 cm^2^) during the storage period. During ripening, guava becomes sweeter due to the degradation of starches and increases in total sugars due to the breakdown of starches into monosaccharides predominately fructose. Another important event in ripening is softening of fruit texture, which is due to the carbohydrate’s hydrolysis (such as cellulose, pectin, lignin, and hemicellulose) by enzymes and which is responsible for cell wall degradation such as polygalacturonase (PG), cellulose, and pectin methyl esterase. Abu-Goukh and Bashir [45] reported PME, PG, and cellulose enzyme activity during ripening of 45–55, 90–120, and 110–125 units/g of guava fruit weight.

### 2.2. Perishability and Storage Life Challenges

Guava is predominately considered as a climacteric fruit, although there are certain varieties of guava that show non-climacteric behavior as well [3,11,21,40,45,46,47,48]. The critical factors for the higher perishability of guava are attributed to an early increase in the respiration rate and biosynthesis of ethylene after the harvest. Guava fruits have shorter shelf life due to higher water content, higher sensitivity, perishability, decay, and higher rate of softening [49]. Jha et al. [19] have reported that for postharvest losses in fruits and vegetables, guava fruits have maximum losses (15.88%). The guava fruits undergo different types of physiological changes during pre- and postharvest, which speed up their rate of respiration, ripening process, ethylene production, and deterioration effects. In addition, the environmental factors along with physical injuries have a negative impact on the guava shelf life and quality attributes like changes in physiochemical and organoleptic properties during the storage. [50]. In order to encounter these losses, the application of postharvest emerging technologies is effective to enhance storage life of guava fruits while maintaining the other quality attributes.

### 2.3. Rate of Respiration

Guava fruit is a biological system that continues to live even after the harvest from the metabolic energy derived primarily due to the process of respiration. Respiration is a catabolic process that occurs in the tissues of fruits and vegetables in which complex macro biomolecules such as carbohydrates, proteins, and fats are broken down into small-molecule such as carbon dioxide (CO_2_) and water (H_2_O), with a release of metabolic energy. For instance, glucose (C_6_H_12_O_2_) present in cells of guava is converted into carbon dioxide (CO_2_) and water (H_2_O) in the presence of oxygen (O_2_).

C_6_H_12_O_2_ + 6 O_2_ → 6 CO_2_ + 6 H_2_O + energy (1)

Respiration is considered to be a critical factor to maintain the postharvest life of guava fruit, as after harvesting of fruit the process of respiration hastens the exhaustion of stored food reserves to maintain cellular organization, membrane permeability, transportation of metabolites, etc., leading to the progression of senescence and quality deterioration [51]. Therefore, a proper understanding of respiration and its rate is critical for developing shelf-life extension technologies for guava fruits. The respiration rate of guava depends on the gaseous exchange of O_2_ and CO_2_ concentrations around the product atmosphere and time of storage at a given temperature. It is usually expressed in the amount of consumption of oxygen and amount of evolution of CO_2_ per unit weight of fruit over per unit time and calculated by using the following equation [6]:

(2) $$R_{O2}=\left[\frac{{\left(Y_{O2}\right)}_{t}-{\left(Y_{O2}\right)}_{t+1}}{∆t}\right]\frac{V_{f}}{W}$$

(3) $$R_{CO2}=\left[\frac{{\left(Z_{CO2}\right)}_{t+1}-{\left(Z_{CO2}\right)}_{t}}{∆t}\right]\frac{V_{f}}{W}$$

where R_O2_ = respiration rate, mL [O_2_] kg^−1^h^−1^; R_CO2_ = respiration rate, mL [CO_2_] kg^−1^h^−1^; Y_O2_ and Z_CO2_ = gas concentrations for O_2_ and CO_2_ respectively; t = storage time in h; Δt = time difference between two gas measurements; V_f_ = free volume of the respiration chamber in mL; and W = weight of the fruit in kg.

Many researchers have reported that guava generally have a moderate rate of respiration, depending upon the temperature and type of cultivator varieties. The respiration rate range for guava fruit is 10–20 mg CO_2_/kg/h at 5 °C and 40–80 mg CO_2_ kg^−1^ h^−1^ at 20 °C [52,53]. Formiga et al. [34] measured the respiration rate of guava cv. Pedro Sato at 21 ± 0.3 °C and RH 77 ± 6% and concluded that respiration rate peak was achieved at 60.02 mg CO_2_/Kg·h after storage period of six days. Liu et al. [23] reported 67.68 mg CO_2_/Kg·h of respiration rate of guava fruit cv. Li-Tzy Bar (LTB) after 8 days of storage. Porat et al. [21] measured the initial respiration rate of guava (cv. Ben Dov and King) 47 mg kg^–1^h^–1^ at 20 °C. After storage of guava, the maximum respiration rate was reached up to 92 mg kg^–1^h^–1^ CO_2_ after 5–8 days of storage in Ben Don variety, and the moderate increase of 58 mg kg^–1^h^–1^ after 8 to 10 days of storage was recorded in King variety of guava fruit. Bashir et al. [41] and Bron et al. [54] reported the respiration rate of guava fruit 34.99 mg CO_2_/kg/h at (22 ± 1 °C 90–95% RH) after 8 days of storage and 42.34 mg CO_2_ kg^−1^ h^−1^ at 21 °C after 156 h of storage, respectively. Guavas predominantly ripen in a climacteric manner, but some cultivator varieties of guavas also ripen in a non-climacteric manner [3,11,21,40,46,47,48]. In climacteric behavior, there is a decrease in respiration rate of guava just after the harvest, which is known as pre-climacteric decline, followed by a sudden increase in rate of respiration, reaching a peak value known as climacteric peak, coinciding with optimum ripening; then, there is a gradual decline in respiration rate, signifying the progression of senescence stage. The sudden rise in respiration rate, also called a climacteric rise, in guava has been studied by various researchers and it is concluded that this is due to biochemical and structural factors [24,55]. The biochemical factors include the rapid increase in production of extra CO_2_ due to the decarboxylation of malic acid; its coupling with phosphorylation hastens the respiration rate. Structural factors, mainly the disorganization and aging of chloroplast structure after harvesting of fruit, result in an increase in enzymatic activity, and biosynthesis of ethylene production hastens the respiration rate [24].

### 2.4. Ethylene Production

Ethylene is known as a natural organic plant hormone that speeds up the ripening as well as senescence process of guava fruit. The precursor for the biosynthesis of ethylene is L-methionine, with the help of enzyme S-adenosyl methionine transferase, 1-aminocyclopropane-1-carboxylic acid (ACC) synthase, and with the help of ACC oxidase for the biosynthesis of ethylene [8,13,55,56,57]. Because guavas are predominantly climacteric, their biosynthesis is stimulated on their own during the early stages of ripening, and other factors such as injury, bruises, heat shock can also increase ethylene production, resulting in faster ripening and senescence. Formiga et al. [34] reported that for ethylene production in guava cv. Pedro Sato stored at 21 ± 0.3 °C, RH 77 ± 6%, no ethylene production was observed up to 4 days of storage and peak value of ethylene production was 12.9 μL of ethylene kg^−1^ h^−1^ on 6 days of storage. Abreu et al. [58] reported ethylene in guava cv. Pedro Sato at 22 ± 1 °C and RH 78 ± 1%, 0.1 µL Kg^–1^/h on initial 4 days of storage; after that, they observed a rapid increase in ethylene production up to 5 µL kg ^–1^/hours of storage of 8 days. Porat et al. [21] noticed the ethylene production in guava fruit (cv. Ben Dov and King) 7.8 mL kg^–1^h^–1^ after 7 days of storage in Ben Dov and 4.1 mL kg^–1^h^–1^ in King variety after 8 days of storage at 20 °C. Liu et al. [23] reported 0.2 × 10^3^ nmol C_2_H_4_ kg^−1^ and 1.37 × 10^3^ nmol C_2_H_4_ kg^−1^ h^−1^ of ethylene production in guava cv.Li-Tzy Bar (LTB) during storage of guava at 20 °C after 2 and 8 days of storage, respectively.

The respiration rate and ethylene biosynthesis are critical parameters for judging the shelf life of guava fruit during storage. The early increase in the rate of respiration and ethylene biosynthesis is associated with the softening of tissues, early maturation, and senescence of fruit, thereby limiting the shelf life of guava fruit. The early setting of senescence due to increased respiration and ethylene biosynthesis can be attributed to depletion of the food reserves inside the cells of the tissues and degradation or dismantling of the cell wall of the tissues, resulting in outward migration of moisture and CO_2_ from the fruit to external environment.

### 2.5. Susceptible to Postharvest Decay

Guava is prone to post-harvest microbial growth mainly fungi causing rots during pre-harvesting, harvesting, storage, and transportation. Guava fruit are mostly attacked by fungal disease known as Anthracnose, caused by *Colletotrichum gloeosporioides* [3,59]. Anthracnose is a kind of latent infection on the surface of fruit skin that remains predominately dormant during the growth stage of fruit but appears as sunken black or brown patches after harvesting of fruit. Embaby and Hassan have isolated six fungi stains from guava, the predominate fungal frequency of *Rhizopus stolonifer* (42.78%), and *Aspergillus flavus* (26.67%) was identified for causing soft rot disease which resulted in post-harvest losses to guava fruit [60]. Amadi et al. have reported the major fungal species responsible for disease in guava were from *Fusarium* (*F. oxysporum*) and Aspergillus genus (*A. niger*, *A. fumigatus*, and *A. parasiticus*) [61]. The microbial invasion of the fruit surface results in not only discoloration of skin color but also degradation of the cell wall of tissues, leading to loss of structural integrity of the fruit. Microbial decay in guava fruits also occurs due to bacterial invasion such as *E. coli*, *B. megaterium*, *M. luteus*, *B. subtilis*, *P. vulgaris*, *B. cereus*, *E. aerogens*, *S. aureus*, *K. pneumoniae*, *S. dysenteriae*, and *S. epidermidis* [62,63].

### 2.6. Chilling Injury

Chilling Injury (CI) is a physiological disorder that occurs when guava is subjected to low-temperature storage (usually less than 10 °C). The symptoms of CI mainly include non-uniform ripening of fruit or fruit failing to ripen, surface pitting, appearance of water-soaked patches, skin and flesh turning brown in color, and increased sensitivity towards fungal decay [64]. The mechanism involved in the development of CI includes (i) over production of reactive oxygen species (ROS), leading to the formation of malondialdehyde byproducts that are responsible for loss of cell membrane integrity; (ii) enzymatic oxidation of phenolic substrates by polyphenol oxidase, resulting in the formation of brown-colored products [8]. Chilling injury results in inducing oxidative stress and malfunctioning of the cell membrane of the fruit tissues, thereby causing a substantial decline in marketable quality of guava, leading to economic loss [65].

Among guava, for measuring the extent of chilling injuries, parameters such as the antioxidant activities of various enzymes such as peroxidase, superoxide dismutase, and building up of malondialdehyde and superoxide anion are estimated during low-temperature storage of fruit [8,22]. Higher severity of CI usually correlates with a decrease in the antioxidant activity of enzymes. The severity of chilling injury on fruit usually increases with storage time.

## 3. Packaging Technologies for Post-Harvest Management of Guava Fruits

**Figure 2:**
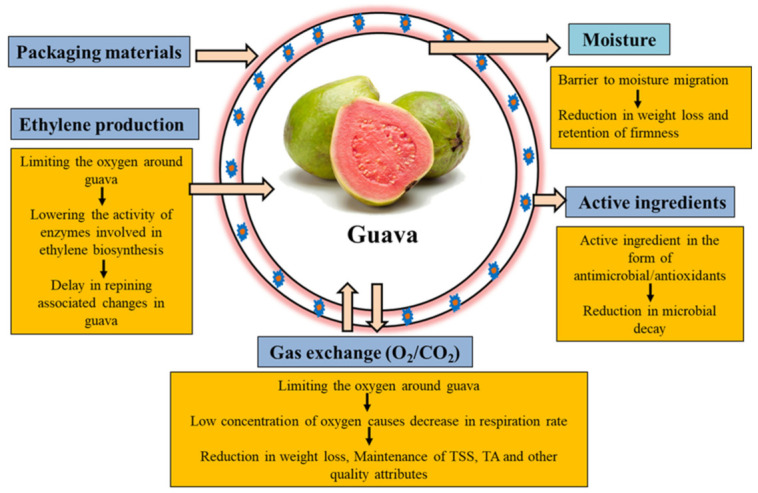
The figure indicating that the application packaging, i.e., edible, active, and composite, has the potential to maintain the postharvest quality attributes, such as color, aroma, firmness, and consumer acceptability by retarding the loss of moisture; reducing ethylene production; and minimizing the lipid peroxidation, respiration rate, and enzymatic and metabolism synthesis due to creation of water and gas barrier between fruit surface and environment.

For the preservation and extension of shelf life of guava fruits during the storage period, various types of packaging have been used, i.e., modified atmosphere packaging (MAP), controlled atmosphere packaging (CAP), edible packaging, composite packaging, antimicrobial/antifungal packaging, and nano packaging. Application of these respective packaging technologies improves the storability of guava fruits during storage due to minimizing oxidation, rate of respiration, physiological loss in weight, and management of ethylene production. Guava fruit had higher respiration and transpiration rate, so to enhance the storage life of guava, the packaging films should have the following characteristics: they should (i) be non-toxic; (ii) have low oxygen permeability; (iii) have high permeability to carbon dioxide; (iv) have a high barrier to water vapor; (v) have good mechanical strength for the protection of fruit; (vi) should not impart any color to the surface of fruit; and (vii) should have no migration or leaching of polymer material onto the fruit [66,67]. Figure 2 presents the mechanism of packaging technologies material on guava fruits during the storage period.

### 3.1. Modified Atmosphere Packaging (MAP)

**Figure 3:**
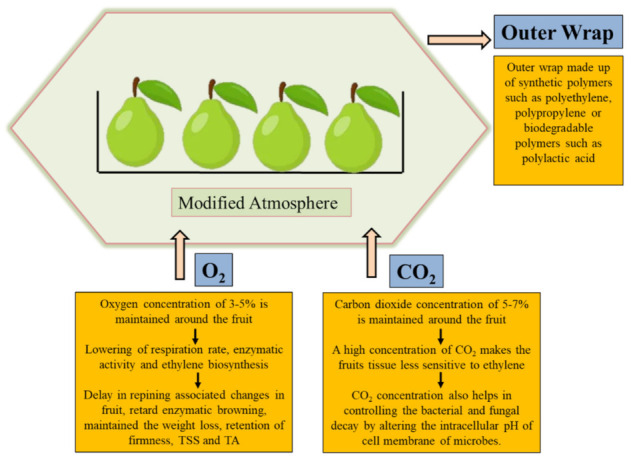
Graphical indication of the mechanism and functions of MAP for improving the shelf life of guava. The low level of oxygen in the MAP packaging is responsible for the retarding the moisture loss, change in color of fruits, and the lower lipid/pigment oxidation. On contrary, the increasing level of carbon dioxide in MAP packaging system of guava fruits has potential to inhibit microbial spoilage while maintaining overall postharvest attributes (modified from: Soltani et al., [73]; Paine and Paine [80]).

In past decade, the use of modified atmosphere packaging (MAP) technology is increasing to maintain and enhance the postharvest quality of fruits and vegetables by utilizing basic principles of permeation and respiration rate to fulfill consumer demand [31,68]. Primarily, the MAP technology was introduced in the 1960s to enhance the shelf life of perishable commodities such as fresh produce by maintaining a suitable environment for commodities and reducing microbial spoilage [69]. It is also considered a novel postharvest technology for the preservation of food and is widely used in developing countries due to lower cost and higher efficiency, utilization of the natural components of air, little environmental impact, and non-toxicity [7,8]. Modified atmosphere packaging (MAP) is known as a dynamic system, which is effective in combatting weight loss, pectin solubilization, and color browning; maintaining firmness; controlling respiration rate; controlling enzymatic activities, and controlling ethylene production of guava fruit during the storage period [24]. Apart from that, it not only enhances the shelf life of fruit and vegetables but also delays the losses of phenolic, flavonoid compounds and antioxidant activity [70,71]. MAP packaging for the guava fruits has depended on the interaction between the respiration rate of produce and packaging materials permeability. The MAP technology is divided into two categories: (i) passive MAP: respiration rate of the produce and permeability of the packaging material are the most important parameters. The respiration rate of fresh produce, consumption of O_2_ is proportional to CO_2_ production in MAP packaging. The total amount of production and consumption of these gases is the same as that passing through the membrane exchange; (ii) active MAP associated with the evacuation of air inside the packaging and replacing atmosphere with desired mixture of gas to accelerate the composition of gases modification to reduce the risk of higher concentration of unsuitable gases [68,72,73]. Figure 3 showed the mechanism and functions of the MAP on fruits and vegetables during the storage period. Badillo and Segura-Ponce reported two types of models, i.e., classic respiration rate and reaction-diffusion model, to understand the relationship between produce and gas permeability [74]. The classic respiration model has included four types of black-box model (exponential, linear, polynomial, and Michaelis Menten kinetic), and reaction-diffusion model included MAP of fruits and vegetables [31]. Furthermore, various researchers reported that the guava fruits stored under modified atmosphere packaging are stored for a long time, with good sensorial and biochemical properties as compared to others. Combrink et al. reported that guava fruit packed in non-perforated polyethylene bags were of a higher quality than guava packed in perforated bags [75]. Pereira et al. enhanced the shelf life of osmotically dehydrated guava fruits for 24 days under storage MAP [76]. Singh and Pal, and Miano and Jokhio, extended the shelf life of guava fruits up to 30 days at 8 °C and 16 °C and 24 °C throughout prevention of physiological loss in weight and other biochemical properties [11,77]. Chandra and Kumar extended the shelf life and maintained the physiochemical, mechanical, and organoleptic characteristics of guava fruits cv. Pant-Prabhat at 25 °C for up to 7 days using MAP packaging technology [78]. Antala et al. and Rana et al. also recorded 21 days shelf life of guava fruits at 10 °C and 7 °C using MAP flushed with 9% O_2_ and 5% CO_2_, and low-density polyethylene (LDPE) [29,31]. Sahoo et al. and Texeira et al. [30] recommended 28 days shelf life of guava fruits stored under polypropylene (PP) MAP [16]. Kumar et al. extended the shelf life of guava fruit cv. *Allahabad safeda* up to 25 days using atmosphere packaging technology at 6 °C storage temperature in polypropylene bag as compared to control (20 days) [79]. They also reported that MAP technology was effective at reducing the chilling injury and controlled titratable acidity of guava fruits. Furthermore, MAP packaging is more effective at preventing weight loss and controlling ripening, as compared to other types of packaging, i.e., cling, shrink, and vacuum packaging [31]. 

### 3.2. Control Atmosphere Packaging (CAP)

Controlled atmospheres packaging (CAP) storage helps to enhance the shelf life of many tropical and subtropical fruits [81]. Storage of fruits in an improper atmosphere may cause increasing fermentation metabolism, resulting in off-flavor development of fruit, ultimately causing fruit to become unacceptable for consumption [82]. CAP storage has limited utility in tropical fruit storage, but it may be very beneficial for marine transport [83]. Increasing demand of tropical fruits, and with variable technological capabilities in developing nations, may provide new opportunities for the implementation of CA storage technique.

The optimized conditions for CA storage of different fruits and vegetables are standardized. Despite its popularity and commercial potential, the reported literature about CA storage of guava fruits is limited.

Kader [81], in their study, suggested controlled conditions of O_2_ (2–5%) and CO_2_ (0–1%) for storage of guava fruits at 5–15 °C. Exposing guava fruits to high levels of CO_2_ (10, 20, and 30%) for short duration had no effect on their respiration rates; however, while ripening, ethylene production was reduced [11]. Storage of guava in the conditions of 10% O_2_ + 5% CO_2_ for 24 h and subsequent storage at 4 °C for 2 weeks resulted in the delay of color development and minimal chilling injury, as compared to the guava stored in the air [84].

CA storage has many beneficial effects on post-harvest quality attributes such as reduced respiration rate and ethylene generation; change in color; softening; retention of vitamins, sugars, and organic acids; and inhibition of some physiological disorders [11,85]. Holcroft and Kader reported that exposing guava to 2–5% O_2_ at 10 °C resulted in delayed ripening of mature-green and partially ripe fruits kept, and the tolerance to elevated CO_2_ was not estimated [86]. Teixeira et al. reported that guava cv. ‘Pedro Sato’ stored at 12.2 °C for 28 days with 5% O_2_ and CO_2_ (1%, 5%, 10%, 15%, and 20%) have not showed any variations in respiration rates; however, the detrimental effects in fruit quality in high-CO_2_ atmospheres (10%, 15%, and 20%) of CO_2_ were observed. Storage of guava in 5% O_2_ + 20% CO_2_ resulted in a sharp decline in firmness, and enhancement in soluble pectin after 14 days [30]. Longer duration of CA storage (low O_2_ and/or high CO_2_) had detrimental effects, including accumulation of ethanol, and acetaldehyde, off-odors, off-flavors, no/delayed ripening after removal from CA storage, and development of injuries [87].

CA storage can also control guava fruit quality loss, which has higher disease susceptibility [83,84]. A lower percentage of oxygen is the prime factor for controlling postharvest fungal development, especially anthracnose and stile rots [88]. Brackmann et al. [89] reported that guava cv. Paluma stored in different CA conditions showed less decay than the ambient atmosphere. The use of nitrous oxide (N_2_O) as a prestorage treatment was reportedly successful in inhibiting the decay development in guava fruits [90]. Singh and Pal [84] found that short-term exposure of guava fruits to very low oxygen (0.1%) and high CO_2_ (40%), at 40 °C for 12 h, resulted in shelf-life extension up to 2–3 days, indicating it was useful for postharvest insect-pest disinfestation purposes. The CA conditions for long-term storage of guava have not yet been defined. The information on the tolerance limits of guava fruits to low O_2_ and high CO_2_ atmospheres is sporadic and inadequate. Table 1 showed the previously conducted investigations to study the effect of MAP/CAP technology on the shelf life of guava fruits during the storage period.

### 3.3. Edible Packaging

Today, due to the rapid use of petroleum-based polymers for the packaging of fruits and vegetables, there is an urgent need for alternative polymers that not only mimic the properties passed by synthetic polymers but also have a very low environmental impact [94,95,96]. In this regard, edible packaging has gained attention in recent times as edible packaging makes use of edible polymers, which not only protect the food from outside environment but can be consumed along with the food. Edible polymers used in edible packaging are made up of polysaccharide, protein, lipids, or their combination and can be made into films or coatings depending upon the fruits or vegetables involved [97]. Edible films and coatings are defined as thin sheets of food-grade material that are fit for human consumption and possess barrier properties (lipid, water vapor, and gaseous transmission) between the food and the external environment, with an ultimate goal of an increase in the shelf life of the food [98,99,100,101]. The words film and coating are used interchangeably in this article; the only difference between film and coating is that polymeric material in films is cast into stand-alone sheets, whereas coatings are directly formed on the product either by dipping or spraying of material [102,103,104]. The mechanism of extending the shelf life of fruits by the use of edible coatings has been demonstrated by various researchers. Edible coatings extend the shelf life of fruits, mainly by providing an extra layer on the surface of fruit, which results in partial closure of stomata and lentils present on the surface of the skin, thereby decreasing the transpiration rate and preventing physiological loss of water from the surface of fruits. Moreover, the coating also fills in the cracks on the surface of the fruits, resulting in fewer chances of microbial growth on cracks or bruises [105].

Guava being a climacteric fruit has a high rate of respiration rate, resulting in a rapid breakdown of complex carbohydrates into CO_2_ and water molecules [3,6,11]. Respiration rate coupled with transpiration rate causes a physiological loss in weight, reduction in nutritional content such as decrease in ascorbic acid, and development of off odor. So, to maintain the quality parameters of guava, edible coatings or films should have low permeability to water vapor and oxygen transmission, to slow down both the respiration as well as transpiration rate at a level that positively affects the shelf life of guava. The permeability to oxygen should not be too low as it can develop anaerobic conditions around the fruit that will lead to the development of off-flavor along with the production of ethanol, thereby limiting the shelf life [24,30]. Edible packaging enhances the shelf life of guava by modifying the gaseous composition around the guava and helps in reducing the ethylene production, transpiration, and respiration rate of guava during storage. Various researchers in recent past have worked on the use of edible films or coatings for the extension of shelf life on fresh guava and demonstrated that the use of edible coatings is effective in reducing weight loss and maintaining freshness and other quality attributes such as total soluble solid content, and ascorbic acid content [26,35,36,106,107]. Edible films for packaging of guava can be characterized into polysaccharide-based edible coatings (including starches from corn, cassava, and potato, and cellulose, hemicellulose, and gums), protein-based edible coatings (including protein-like casein, zein), lipid-based edible films (including oil and waxes), and their composite films (combination of polysaccharide or protein with lipid) [34,38,97,108,109,110,111,112].

#### 3.3.1. Polysaccharide-Based Packaging

Polysaccharide is abundantly available in nature and usually made up of smaller repeated carbohydrate units (monosaccharide) that are linked to each other with a glycoside bond. Polysaccharides that are widely used for making edible coatings are starches (corn, wheat, cassava, and potato) and cellulose derivatives (methyl cellulose, carboxyl methyl cellulose, hemicellulose, pectin, alginates, gums like guar, xanthan, carrageen, and agar-agar) [97]. Polysaccharide-based edible films are nontoxic and possess great mechanical strength, good barrier properties to oxygen due to the presence of hydrogen bonding, and form a tightly packed network between the molecules of polysaccharide. The selective barrier property to oxygen and carbon dioxide shown by polysaccharide-based films helps in maintaining the respiration rate of the guava, thereby increasing the shelf life of guava. Some of the starch-based edible films for extending the shelf life of guava are shown in Table 2. The main drawback of polysaccharide-based edible films is that it is hydrophilic and shows poor resistance to water vapor [36,113,114,115]. Films made from polysaccharides usually have a very high-water vapor transmission rate, resulting in evapotranspiration from the fruit surface, leading to shrinkage and physiological loss of weight. Francisco et al. [116] conclude that edible coatings made from 25% acetylated cassava starch (ACS) and 75% hydroxyethyl cellulose (HEC) is effective in extending the shelf life of guava to 13 days with minimum damage to vitamin c content along with better retention of fruit firmness. Quirino et al. [112] claim that 4% cassava starch coating on guava maintained the shelf life up to 12 days of storage at 25 ± 1.0 °C without significant loss of weight and firmness. Moreover, coated guava shows higher retention of ascorbic acid and TSS, compared to uncoated fruits. Krishan and Rao reported that coating of guava fruit cv. *Allahabad safeda* with 1% chitosan solution helps in extending the storage for 21 days at 12 °C [117].

#### 3.3.2. Lipid-Based Packaging

Lipids are the complex organic compound made up of esters of fatty acids and mainly include fats and oil. Lipid-based coating in the form of waxes is widely used for coating fruits to provide surface gloss as well as to provide a barrier to moisture. The coating or films made from lipids are hydrophobic and possess excellent barriers to water vapor migration [118,119,120]. The only drawback of using lipid-based films for coating climacteric fruit such as guava is that sometimes due to their hydrophobic and excellent barrier to oxygen and carbon dioxide, they cause anaerobic respiration, resulting in the production of off-flavor and early senescence of the fruit [121]. Madhav et al. [122] claimed that coating of guava with vegetable wax (1:4 *v*/*v*) extended the shelf life for 12 days followed by 2 days table life at ambient condition (20 °C), without significant loss of weight and better retention of firmness and TSS (total soluble solids). Zambrano reported that coating of guava with 10% and 20% with cactus mucilage maintained the quality of guava at 16 days at 10 ± 1 °C, without significant loss in weight and firmness in coated guava [123]. Ruzaina et al. also claimed that coating guava with palm stearin and palm kernel olein (1:1) keeps the shelf life of guava for 21 days at 20 °C and 30 days at 10 °C without significant loss of quality [109]. Zahid et al. extended the shelf life of guava fruit by 8 days using bee wax-based edible coatings at 25–27 °C, RH 80–90% [108].

#### 3.3.3. Composite Packaging

Composite packaging and material are made up of a combination of polysaccharides or protein, along with lipids for fulfilling the limitations posed by individual polymer films and to enhance the functionality of films [111,124,125]. Composite films or coatings are mainly used for extending the shelf life of fresh produce (guava) as they maintain the desired properties of permeation of gases and water vapor between guavas and the surrounding environment [35,37,110,126]. Some of the composite films previously developed by the researchers are summarized in Table 2.

**Table 2:** Composite edible packaging to enhance the shelf life of guava fruits.

Matrix	Guava Variety	Effect on Quality Parameter	Shelf Life	References
Gum Arabic (10%) + garlic extract (20%)	*Gola*	Reduction in loss of weight, skin browning; retention of ascorbic acid with lower value of total sugars; and increase in flavonoid content.	15 days at 25 ± 3 °C	[37]
Agar (4%) + pomegranate seed oil (0.4 mL/L)	*Paluma*	Reduction in weight loss; no significant change in carotenoid content; Total soluble solid, skin color was maintained without excessive lose	10 days at 10 °C and 40% RH	[36]
Carboxymethyl cellulose (2.7%) + stearic acid (2.1%) + lecithin (3.2%) + date pit oil (2%)	*-----*	Reduction in weight loss; retention of ascorbic acid, firmness, TSS, and titratable acidity, compared to control sample.	16 days at 25 °C	[107]
Tamarind seed Powder (0.05%) + Beeswax (1%) + sunflower oil (5.5%)	*-----*	Reduction in weight loss; retention of ascorbic acid, firmness, TSS, and titratable acidity, compared to control sample.	13 days at 30 ± 2 °C and 21 days at 25 ± 1 °C.	[127]
Tamarind starch (3%) + pomegranate seed oil (0.24 mL/mL)	*Paluma*	Reduction in loss of weight; better retention of firmness as than control sample; delay in ripening of fruit.	21 days at 10 ± 2 °C and 80 ± 5% RH	[35]
Arrowroot starch (2%) + pomegranate oil (0.3%)	*Paluma*	Reduction in respiration rate, causing delayed ripening of fruit; retention of firmness, ascorbic acid; reduction in weight loss as compared to control sample.	20 days of storage the temperature of 10 ± 2 °C with 85 ± 5% RH	[126]
Chitosan (1%) + poly-vinyl-pyrrolidine (1%) + salicylic acid (2 mM)	*Banati*	Coated guava showed reduced enzyme activity of polyphenol oxidase, cell wall degrading enzymes; retention of skin color, and firmness; and reduction in weight loss as compared to control samples.	15 days at 27 ± 1 °C and 48 ± 2% RH	[128]
Hydroxypropyl methyl cellulose (5%) + beeswax (20%)	*Pedro Sato*	Reduction in loss of weight; retention of firmness; and decrease in L* value and hue angle as compared to control sample.	8 days at 21 ± 0.3 °C and 77 ± 6% RH	[34]
Jackfruit seed starch (2%) + chitosan (2%) + alignate (2%)	*Paluma*	Reduction in loss of weight; retention of firmness; and decrease in L* value and hue angle as compared to control sample.	22 days at 10 ± 2 °C e 80 ± 2% RH	[106]
Hydroxypropyl methyl cellulose (1%) + palm oil (0.3%)	*Lalit*	Decrease in enzyme activity of polyphenol oxidase (PPO) and peroxidase (POD); reduction in loss of weight, color value, and retention of firmness.	12 days 24 ± 1 °C and 65 ± 5% RH	[129]
Chitosan (3%) + 4% palm stearin: palm kernel olein (75:25)	*----*	Coated samples showed reduction in respiration rate and ethylene production, and reduction in loss of weight and retention of firmness and TSS as compared to uncoated samples.	31 days at 5 ± 2 °C	[110]
Cashew gum (1%) + carboxymethylcellulose (2%)	*Kumagai*	MRI studies of coated guava reveled that after 8 days of storage, there is surface tissue decay; retention of firmness and ascorbic acid; reduction in weight loss as compared to uncoated fruits.	08 days at 25–28 °C and 76.0 ± 12.4% RH	[26]

### 3.4. Antimicrobial/Antifungal Packaging

Guava is prone to fungal decay during preharvest as well as after harvesting and storage. For inhibition of fungal microflora, the use of chemical fungicides such as benomyl, carbendazim, triforine, prochloraz, and mancozeb is very common, but studies show that these chemical fungicides leave chemical residues on the surface of fruit that are considered potent carcinogens and are even banned in some countries. In the last few decades, natural antioxidant and antimicrobial agents, produced from fruits and vegetables, and their wastes, have been incorporated in edible packaging material to enhance the properties of materials and respective fruits [130]. These antioxidant agents influence the efficacy of materials and enhance the surface properties of fruits, inhibiting microbial and fungal contaminations [67] due to the presence of higher contents of secondary metabolites, including phenolic content and other tannin groups. Various types of plant-derived essential oils and extracts can be used as natural antioxidant and antimicrobial agents to protect fruits from microbial contaminations; this protection is likely conferred by the presence of hydroxyl groups, which help in the deactivation of PPO/POD enzymes [131]. Essential oils are volatile natural compounds extracted from aromatic plant materials that have antioxidant and antimicrobial activities. This group of natural antimicrobials with GRAS (Generally Recognized as Safe) status is used in the food industry for controlling and inhibiting undesirable micro flora for the effective preservation of food. Lourenço et al. [132] briefly explained this in their review paper about the plant origin source of antioxidant compounds and their utilization in food industries. Many researchers have reported that the incorporation of antioxidant, anti-browning, and antifungal agents in edible coatings and films can prevent microbial decay, fungal decay, lipid oxidation, sensory characteristics, and color changes in the fruits [133,134]. Soares et al. [135] reported that the application of cassava-based antimicrobial edible coatings helps to prevent and enhance the shelf life of guava fruits by reducing the growth of microbes. Murmu and Mishra showed that gum-Arabic-based edible coatings formulation with sodium caseinate and enriched with natural antimicrobial agents such as *Tulsi* extract enhanced the shelf life of guava fruits for 7 days as compared to 4 days of control at 28 °C [17]. The application of optimized and recommended formulation of edible coatings 5 g/100 mL (gum Arabic), 1 g/100 mL (sodium caseinate), and 2.5 mL/100 mL (*Tulsi* extract) was found to be potentially effective at preventing microbial decay, and other postharvest quality of guava. Othman et al. [136] incorporated (0.050 g/100 mL coating solution) the sunflower and marjoram essential oil in carboxy-methylcellulose and alginate-based edible coatings to investigated their effect on guava fruits during storage for 28 days and reported that the application of essential-oil-based antimicrobial edible coatings ensured overall quality, including texture, mass loss, appearance, and microbial safety (mold and yeast) of guava fruits during storage. Elabd enhanced the shelf life of guava slices using potato starch and chitosan based edible coatings by maintaining postharvest quality and reducing microbial decay and enzymatic activity. He reported potato starch and chitosan as natural antimicrobial agents for the preservation of guava slices and extension of the shelf life of guava slices, during storage [137]. Murmu and Mishra used different concentrations (1%, 2%) of cinnamon and lemon grass essential oil in Arabic-gum- and sodium-caseinate-based antimicrobial edible coatings to improve the postharvest shelf life of guava fruits [7]. The application of edible coatings enriched with essential oils was found to enhance the shelf life of guava fruits by up to 40 days by reducing browning and degradation of antioxidant activity, increasing retention of phenolic and flavonoid compounds, and reducing the rate of sugar loss. Nair et al. prolonged the shelf life of guava fruits using chitosan- and alginate-based edible coatings enriched with pomegranate peel extract. The incorporation of pomegranate peel extract in edible coatings helps to maintain the phenolic, flavonoid, and antioxidant activity of guava fruits during storage for 20 days at low temperatures [33]. Arroyoa et al. investigated the effect of chitosan- and alginate-based antimicrobial edible coatings enriched with ZnO nano materials on guava fruits and enhanced the shelf life of fruits with degradation of microbial activity and rotten index; they also protected the guava fruits against excessive mass loss and retardation of physiochemical changes [138]. Etemadipoor et al. investigated the effect of gum-Arabic-based edible coatings enriched with natural antimicrobial agents such as cinnamon essential oil and improved the storability and postharvest quality of guava during storage for 28 days at 10 ± 1 °C, 90–95% RH [25]. They found that edible coatings enriched with essential oil were effective in reducing ripening rate and respiration rate, maintaining color, and maintaining antioxidant properties of guava fruits. They also recommended an effective formulation of edible coatings with 10% gum Arabic and 1% essential oil of cinnamon to improve the storability of guava fruits. Etemadipoor et al. reported the incorporation of essential oil and oleic acid in gum-Arabic-based edible coatings has potential and is effective in increasing the storability of guava and maintaining the postharvest quality. The coating formulation was also found to be effective in preventing chilling injury, delaying browning, maintaining firmness, and ameliorating changes in bioactive compounds of guava fruits during the storage at 10 ± 1 °C, RH-90% for 28 days [38]. Cid-Perez et al. studied the antifungal activity of *Poliomintha longiflora* oil and reported that MIC (minimum inhibitory concentration) of *Colletotrichum gloeosporioides* was achieved with 0.8–1.0 (g/L) concentration of essential oil [139]. Abd-Aiiam and Haggag reported that basil essential oil at 250 (µg/mL) concentrations is effective in inhibiting *Colletotrichum gloeosporioides* [140]. Viuda-Martos reported that the concentration of oregano essential oil at 4 mL/18 mL culture medium inhibited the the % growth of *A. flavus* 100%, whereas for achieving the same 100% growth inhibition of *A. flavus* the clove and thyme the concentrations were 6 mL/18 mL and 8mL/18 mL culture media, respectively [141]. The exploration of the incorporation of essential oils and plant extracts for edible packaging of guava is very recent for extending the shelf life of guava by preventing fungal infections such as Anthracnose. Thyme-, clove-, lemongrass-, and oregano-coated essential oil edible coatings show a strong antifungal effect on microbial growth and help extend the shelf of guava. Apart from inhibiting or preventing microbial growth, the use of essential oil in edible coatings also improves water vapor permeability and gaseous transmission (O_2_ and CO_2_). Table 3 summarizes the effect of essential oil rich edible packaging on the shelf life of guava fruits.

### 3.5. Nano-Based Packaging

During the development of composite films, there is no proper blending of polymers, which results in the development of films with poor barriers and mechanical properties, which results in non-uniform distribution coatings on fresh produce. To overcome these drawbacks, in recent times concept of nano technology has been used for designing edible coatings for enhancing functional properties such as homogeneity or blending of polymers, to attain uniform morphological characteristics, better permeability to water vapor, and gaseous exchange. Nano particles such as zinc oxide (ZnO) and solid lipid nano particles (SLN) are used in edible-coating matrices to improve the functionality of edible coatings in increasing the shelf life of guava. Garcia-Betanzos et al. applied solid lipid nanoparticles/xanthan gum-based coating on guava cv. Media China and extended the shelf life of fruits at 10 °C for 32 days [145]. Nano-particles-based coatings were found to be effective at controlling the nutritional and other biochemical properties of guava fruits. González-Reza et al. also investigated the effect of solid lipid nanoparticles on fresh-cut guava fruits. They reported that the lipid-nano-particle-based coating was effective in extending shelf life of guava fruits, control browning index, and maintaining firmness and sensory characteristics during the storage period for 22 days (at 7 °C and 85% of RH) [28]. Arroyo concludes that the addition of 1% nano ZnO in chitosan (100%) or chitosan (90%) blend with alginate (10%)-based edible coatings maintains the shelf life of guava for 15 days at 21 ± 1 °C and 80 ± 2% RH, compared to control sample (without coating) of 7 days [138]. The increase in shelf life is attributed to the addition of nano ZnO, which not only improved the water barrier properties of the coating but also exerted antimicrobial action during storages of the guava. Zambrano-Zaragoza et al. reported that SLNs (solid lipid nano particles) made from Carnauba wax (65 g/L) are dispersed with xanthan gum (4 g/L) and polyethylene glycol (5 g/L) to form a filmogenic dispersion [146]. Gad and Zagzog claim that edible coatings made from xanthan gum (1%) along with 0.2% chitosan nano particle could extend the shelf life of guava by 35 days at 8 ± 1 °C and RH 85–90%, followed by shelf life of 5 days at 20 °C [147]. The application of dispersion on guava by dipping method helped maintain the quality of guava for 30 days at 10 °C and 85% RH, followed by 5 days at room temperature (25 °C). The addition of SLN produced a homogenous coating on guava, which resulted in reduced respiration and transpiration rate from the skin of guava, leading to an extension of shelf life.

## 4. Effect of Packaging Technologies on Postharvest Characteristics of Guava Fruits

### 4.1. Physiological Weight Loss (PLW)

Physiological loss in weight (PLW) is considered a major factor for the short shelf life of the guava fruits since it causes shriveling and browning. The weight loss of the fruits has been gradually decreased in both control and treated (packaged) fruits, but a significantly higher loss in physiological weight has been found in control as compared to treated [148]. Hernández-Muñoz has reported that PLW primarily reflects the moisture evaporation and respiration rate between the surrounding environment and fruit tissue due to influence of postharvest treatments [149]. The PLW of guava fruits is generally attributed to loss of the free and bound moisture through respiration and evapotranspiration. The higher rates of respiration and moisture evapotranspiration are generally caused by weight loss in guava fruits and affect the shelf life and quality parameters such as firmness and visual appearance. [150]. Various researchers reported that the application of postharvest management and packaging materials (MAP, GAP, edible packaging, nano-coating, etc.) treatment improved the shelf life of guava fruits and reduced the loss in physiological weight [24,33,147]. Singh and Pal investigated the effect of control atmosphere packaging on weight loss of guava fruits cv. Lucknow-49′, ‘Allahabad Safeda’, and ‘Apple Color’. They reported the application of control atmosphere (2.5, 5, 8, and 10 kPa O_2,_ with 2.5, 5, and 10 kPa CO_2_) was effective in the prevention of physiological weight loss in guava fruits during the storage period at 8 °C [11]. Zambrano-Zaragoza et al. The authors of [146] reported the prevention and beneficial effect of the lipid nanoparticles on the weight loss of guava fruits during storage period for 30 days at 10 °C, due to minimizing the respiration rate and barrier against water loss. Previous researchers—Jacomino et al. [151]; Sunjka et al. [152]; and Mangaraj et al. [24] reported that the MAP is a potential technology to prevent physiological loss in weight of guava fruits by controlling the respiration, oxidation, and water transpiration. Krishna and Rao [117] investigated the effect of chitosan-based edible coatings on guava fruits cv. *Allahabad safeda* and found that the application of chitosan-based edible packaging had the potential to retard the weight loss of guava during the storage period (28–32 °C and 32–41% RH), and extend the shelf life for 7 days, probably due to delaying ripening process, reduction of respiration rate, and maintenance of the rigidity of guava fruits.

### 4.2. Respiration Rate

Guava is a predominately climacteric fruit that continues to respire after harvesting and shows a climacteric peak in a short period of usually 24–48 h depending upon the temperature of storage conditions. The early onset of climacteric peak results in an increase in respiration rate, and other biochemical changes such as shifting of skin color, increase in TSS, decrease in acidity, and firmness of the fruit [3,21,153]. The increase in respiration rate results in the breakdown of complex biomolecules, in either presence or absence of oxygen, into byproducts such as water and carbon dioxide, along with metabolic heat. Oxygen plays a critical role in the respiration rate of guava because limiting the concentration of oxygen can cause a reduction in respiration. The aim of packaging technologies such as edible films and MAP is to delay the onset of climacteric peak by modifying the atmosphere surrounding the guava, thereby reducing the respiration rate, ultimately leading to increased shelf life. In Pedro Sato guava coated with hydroxypropyl methylcellulose and beeswax stored at 21 ± 0.3 °C and relative humidity of 77 ± 6%, there was a reduction of 12% in respiration rate of coated guava as compared to control samples on the sixth day of storage [34]. Murmu and Mishra reported that edible coatings of guava with Arabic gum with sodium caseinate and Tulsi extract stored at 7 days of storage at 28 ± 2 °C resulted in a decrease in the average O2 consumption rate of 10.08 cm^3^/kg h in coated samples as compared to control samples (24.49 cm^3^/kg h), indicating that a reduction in respiration rate led to an extension of shelf life from 3 days (uncoated) to 7 days for coated samples [17]. A similar study was conducted by Nair et al. who concluded that edible coatings made from chitosan incorporated with pomegranate peel extract reduced the respiration rate of guava by 28.6% as compared to uncoated samples. The decrease in respiration rate was attributed to the creation of semi-permeable barrier that modified the internal gaseous composition O_2_ and CO_2_ in such a way that it had a positive effect on respiration rate that delayed the ripening process [33].

### 4.3. Ethylene Production

Ethylene (C_2_H_4_) is a plant hormone that induces ripening and is responsible for the activity of various enzymes that brings physiological and biochemical changes to fruit. Because guava is a climacteric fruit, the ethylene production peak is attained during the initial stages of ripening, and in most of the guava cultivars it does not coincide with the respiratory peak [11,21,47,154]. For biosynthesis of ethylene, oxygen is the main substrate that helps the activity of enzymes such as ACC synthase and ACC oxidase, for the synthesis of ethylene hormone [155,156,157]. In the presence of limited oxygen, the biosynthesis of ethylene slows down due to a decrease in respiration rate. Packaging technologies such as edible films and MAP work by limiting the oxygen by creating a semi-permeable barrier to gaseous exchange that results in suppression of respiration rate, which ultimately enhances the shelf life of guava. Formiga et al. reported that in Pedro Sato guava coated with edible coatings, no peak of ethylene was observed, whereas in the uncoated sample the ethylene peak (12.9 μL of ethylene kg^−1^h^−1^) was observed after six days of storage [34]. Vishwasrao and Ananthanarayan, reported that edible coating of hydroxypropyl methyl cellulose and palm oil delays the ethylene peak from 3 days for the control sample to 6 days for coated sample stored at 24 ± 1 °C and 65 ± 5% RH [129]. The delay in ethylene production contributes to an increase in the shelf life of guava from 9 days (uncoated sample) to 12 days for coated samples. Many researchers have reported that edible coatings are effective in delaying ethylene production during storage of guava [22,158,159].

### 4.4. Color

The color of the guava skin generally varies from light green to yellow depending upon the stage of maturity, and pulp color can be white, creamy, pink, or light red depending upon the guava variety. Generally, during the ripening stage there is a change in skin color of guava from green (mature green stage), to greenish-yellow, to yellow of 40–70%, and to yellow of more than 70%. The quantitative estimation of fruit color is done by using colorimeter technique color models such as RGB (red, green, and blue) and CIELAB (L*, a*, b* values) models [24]. In CIELAB models, L* denotes lightness and its values vary from 0 (black color) to 100 (white color); a* and b* values vary from +60 to −60, negative value of “a” denotes the color change from blue to green, while a positive value of b denotes the yellow color. L*, a*, and b* of Bandipur guava at a mature green stage were reported as 57.83 ± 5.16, −17.58 ± 2.59, and 39.41 ± 3.47, respectively. The change in color from green to yellow during ripening is generally attributed to changes in pigment content, such as a decrease in chlorophyll content and an increase in carotenoid content. Siqueira et al. reported that during ripening, the color of plume guava stored at 27 °C changed from green to yellow due to a 72% reduction in chlorophyll content and 400% increase in carotenoids content [160]. Moreover, the enzymatic activity of polyphenol oxidase in the presence of oxygen leads to browning of skin, which affects the color of fruit [38]. Packaging technologies such as edible coatings and modified atmospheric packaging have shown the retention of green color during storage of guava [24,96]. Etemadipoor et al. reported that edible coatings helped to maintain the green color of guava by slowing down the rate of chlorophyll degradation, which was evident from values of L* and a*, which were not significantly affected by the use of the edible coatings [25]. Germano et al. also reported that coating of guava with edible films resulted in a decrease in chlorophyll content by 19.80%, whereas in uncoated samples the reduction in chlorophyll was 86.94% [161]. de Aquino et al. and Santos et al. confirmed that edible coatings confer better retention of green color of guava fruits as compared to uncoated [144,158].

### 4.5. Total Soluble Solid (TSS)

Total soluble solids (TSS) are important indicators of the sweetness of the fruit and are generally correlated with fruit maturity and ripeness. During the process of ripening, there is an increase in the TSS content due to the hydrolysis of starch into simpler sugars such as fructose and glucose [3,38]. Because guava is a climacteric fruit, there is an increased rate of respiration with a high rate of metabolic activity during its storage, resulting in the acceleration of starch hydrolysis. Moreover, the physiological loss of water during storage of guava, there is oxidation of organic acid which helps in increasing the TSS of guava fruit [129,162]. Various packaging technologies such as edible packaging and modified atmospheric packaging are effective at reducing the increase in TSS content of guava fruit by reducing the rate of respiration and providing a barrier to moisture loss [7,24,158]. Etemadipoor et al. reported that edible coatings of guava reduced the increase in TSS stored at 10 °C. The maximum increase in TSS occurred in uncoated guava (48.79%), whereas in coated guava level of increase was only 29.93% [38].

### 4.6. Titratable Acidity (TA)

Titratable acidity (TA) is another parameter that affects the eating quality of fruit. Titratable acidity measures the amount of acid present in the fruit that is titratable. Ascorbic acid, malic acid, and citric acid are the predominant organic acids present in guava, and these organic acids are used as the main substrate for respiration and other metabolic activities. During storage of guava, there is a continuous increase in respiration rate, which results in loss of organic acid there due to a decline in TA [163]. Edible coatings can minimize the losses in TA during the storage of guava. Etemadipoor et al. [38] reported that edible-coated guava effectively maintained the TA 2.93 times more than the TA of uncoated guava stored at (at 10 ± 1 °C and 90% relative humidity) for 28 days. Vishwasrao and Ananthanarayan also showed that the TA of coated samples (0.89) after 9 days of storage is almost the same for uncoated samples (0.87) after 6 days of storage [129]. The reason for minimizing the TA losses in edible-coated guava is the creation of a semipermeable membrane by the coating material around the fruit. The coating material allowed the selective permeation of oxygen molecules that resulted in a reduction in respiration rate and preventing the oxidation of organic acids during storage [164,165].

### 4.7. Firmness

Firmness is an important quality parameter for determining the intactness of the cell wall constituents and the overall acceptability of fruit. During the ripening process or storage of fruit after optimum maturity, there are gradual decreases in firmness due to the dissolution of cell wall constituents and alterations of pectin fractions by an increase in enzymatic activity of pectin hydrolyzing enzymes such as polygalacturonase (PG) and pectinesterase (PE), resulting in a reduction in cell-to-cell adhesion and solubilization of pectin [7,166,167,168,169]. Various researchers have reported the different methods for measuring the firmness of guava fruit, and the most widely used methods are texture profile analysis (TPA), puncture strength, and compression test [24,38]. The firmness of fruit depends upon the stage of maturity and the variety of fruit and is influenced by different packaging technologies such as edible coatings and MAP. Etemadipoor et al. reported that edible coatings made from 10% gum Arabic enriched with 1% cinnamon are more effective at maintaining fruit firmness (40.27 N) after 28 days of storage at 10 °C than the uncoated samples (19.2 N) [25]. Germano et al. claim that edible coatings made from Galactomannan-carnauba wax maintained the firmness of guava from its initial value of 65.06 ± 4.87 N to 56.46 ± 15.01 N after six days of storage at ambient temperature 25 °C, where, as in uncoated samples during the same period, the firmness value was reduced from 65.06 ± 4.87 N to 6.72 ± 0.86 N. The decrease in firmness value is attributed to the increase in enzymatic activity of lipid peroxidation (LP), increase in PME activity, and decrease in PG activity, which resulted in loss of cell membrane fluidity, integrity, and solubilizations of pectin present in the cell wall [161]. Murmu and Mishra reported that edible coatings of Arabic gum with sodium caseinate and Tulsi extract maintained the firmness (37 ± 7 N) after seven days of storage at 28 ± 2 °C, compared to uncoated samples (14.81 ± 7.23 N) [17]. Various researchers also reported that edible films are effective at maintaining the firmness of guava during storage by maintaining the barrier to water vapor and decreasing respiration and ethylene production. As enzyme activity is dependent upon oxygen, the change in concentration of oxygen around fruit can effectively help in the retention of firmness [155,170].

### 4.8. Ascorbic Acid (AA)

Guava is considered one of the richest sources of vitamin C or ascorbic acid (AA) and is vital for human beings because of its inherent immune protection properties. The AA content in guava varies from 100–300 mg/100 g or even more depending upon the guava variety. The ascorbic acid content in guava is maximum at the mature green stage and subsequently starts decreasing during ripening or storage, and the decrease in ascorbic acid is due to the oxidation of ascorbic acid into dehydroascorbic acid by the action of ascorbic acid oxidase [7,16]. The oxidation of ascorbic acid can be minimized by use packaging such as MAP and edible coatings on guava because these technologies aim at limiting the oxygen concentration around the product, which helps decrease the oxidation of ascorbic acid by ascorbate oxidase. Etemadipoor et al. reported that coatings of guava with 10% gum Arabic with 1% cinnamon essential oil increased the retention of ascorbic acid during storage of guava at 10 °C for 28 days [38]. Murmu and Mishra reported that edible coatings can effectively retain the ascorbic acid content (45–67% retention) better than the uncoated sample (29–33%) stored at 4–7 °C & 80% RH. Mangaraj et al. [24] also demonstrated that reducing the oxygen concentration to as low as 5% in the package can better retain ascorbic acid, compared to unpacked guava stored at ambient temperature [8].

### 4.9. Microbial Decay

Microbial decay in guava fruits mainly occurs due to *E. coli*, *M. luteus*, *P. vulgaris*, *E. aerogens*, *B. subtilis*, *B. megaterium*, *B. cereus*, *S. aureus*, *S. dysenteriae*, *K. pneumoniae*, *S. epidermidis* [62], *Phoma* spp., *Penicilium* spp., *Aspergillus* spp., and *Colletotrichum* spp. [63], which cause green/blue mold rot, grey/brown rot, aspergillus rot, mucor rot, phomopsis rot, rhizopus rot, and soft rot, respectively. Sandarani et al. reported that the application of MAP technology can reduce microbial growth and deterioration effects on guava fruits during the storage period [171]. Lima et al. investigated the effect of MAP technology (PVC) on guava fruits cv. Paluma reported that the application of MAP can control the growth of viable mesophylic microorganisms and prolong the shelf life of guava fruits for up to 6 days at 3 °C [5]. Arroyo et al. investigated the effect of active antimicrobial edible coatings with nanoparticles on postharvest physiology of guava fruits at 21 ± 1 °C and 80 ± 2% RH and found out that the incorporation of the active ingredients in edible coatings controls the growth of microbes, which helps to prolong the shelf life of guava fruits [138]. Othman et al. used sunflower and marjoram essential oils as active ingredients with carboxymethyl cellulose- and alginate-based edible coatings to improve the microbial stability and prolong the shelf life of guava fruits during a storage period of 28 days [136]. They also found that the application of essential-oil-enriched edible coatings was effective at controlling the growth of mold, yeast, psychrophilic bacterial counts, and total bacterial counts in guava fruits during the storage. Many researchers have reported that applications of postharvest packaging technologies, such as MAP, CAP, edible packaging, antimicrobial packaging, and nano packaging, have the potential to provide microbial safety for guava fruits and prolong shelf life by reducing deterioration effects and chilling injuries [7,33].

### 4.10. Chilling Injury

Chilling injury of the guava fruits can cause them to adopt a mature green color and can cause external and internal browning (skin and flesh) of the fruits during the storage period [172]. Chilling injury of the guava fruits is directly linked to the changing of cell membranes in these fruits [173]. Recommended cold storage temperature for guava fruits has been reported as 10 °C, to protect the guava fruits from chilling injury [174]. The direct effect of the low storage temperature can responsible for the loss of integrity of the fruits; increase the lipid oxidation process and levels of reactive oxygen species (ROS); and have an impact on the quality and safety of guava, as well as economic consequences [175]. The chilling injury symptoms of guava fruits include post-ripening decay and damage [176]. Apart from that, various postharvest packaging technologies and treatments have been applied previously to protect guava fruits from chilling injury and prolong their shelf life [173]. The applications of postharvest management and packaging technologies can protect guava fruits from chilling injury, and many researchers have proved this. Antala et al. [29]; Kumar et al. [79]; and Murmu and Mishra have reported that MAP technology has the potential to prevent guava from chilling injury at low temperatures and minimize lipid peroxidation and browning [8]. Many researchers have confirmed that the application of edible coatings and films with active ingredients, i.e., essential oils, plant extracts, and nano materials, has a beneficial impact on guava fruits and helps to prevent chilling injury and oxidative stress [33,38,129,136].

### 4.11. Sensory Characteristics

Sensory characteristics are important parameters for judging the quality of fresh guava and its marketability and acceptability to consumers. Fresh guava is characterized by its sweet taste and peculiar aroma due to the presence of volatile compounds such as (Z)-3-Hexenal and cinnamyl acetate [177]. Its sensory characteristics such as taste, aroma, and texture depend on the stage of maturity, varieties, and storage conditions of guava. The effect on the sensory properties of the guava fruits is studied by various researchers, and it is concluded that edible coatings are effective at maintaining the sensory properties of guava during storage period [8,37,178]. A study by Anjum et al. demonstrated that edible coatings made from gum Arabic and ginger scored higher sensory ratings for taste (6.66) and aroma (7) on 9-point hedonic scale than uncoated samples (2.66 taste score and 3.33 aroma score). In another study, de Olivera et al. pointed out that edible coatings of chitosan with lemongrass essential oil scored higher sensory number on hedonic scale for color, firmness, and overall acceptability than uncoated samples after 5, 10, and 15 days of storage. The increased preference for edible-coated fruit is probably due to the delay in the ripening-associated changes and maintenance of textural properties [179]. Olivera et al. also observed the positive effect of edible coatings on the sensory characteristics of guava and reported that for measuring the purchasing intention of guava, 90% of sensory panelist gave a score of 4 or 5 (probably would buy and would certainly buy) for the edible-coated guava and 78% of panelist gave a score of 1 or 2 (certainly would not buy and probably would not buy) for the uncoated samples after 15 days of storage [180]. 

The literature review on packaging technologies have clearly demonstrated the positive effect on the shelf life of the guava fruit at different storage condition. In nutshell, the main outcomes of packaging technologies on the postharvest characteristics of guava fruits are listed in Table 4.

## 5. Conclusions

The use of postharvest technologies such as modified atmosphere packaging (MAP), controlled atmosphere packaging (CAP), edible packaging, composite packaging, antimicrobial/antifungal packaging, and nano packaging, to enhance and delay ripening, respectively, can significantly reduce postharvest losses of guava fruit throughout the storage and value chain. Controlling the respiration rate, reducing weight loss, minimizing lipid oxidation, and maintaining the organoleptic properties of guava fruit is useful, especially during peak season and storage. The proper use of postharvest technologies such as modified atmosphere packaging (MAP), controlled atmosphere packaging (CAP), edible packaging, composite packaging, antimicrobial/antifungal packaging, and nano packaging will lead to an increase in the safety of guava fruits and adherence to quality standards for international and national markets. The advantage of using these postharvest technologies on guava fruits has been emphasized with strong research evidence. Despite the advantages of postharvest technologies, only a few packaging systems are commercialized due to food safety regulations, consumer acceptance, and high cost. Therefore, more research is needed to develop an effective packaging system for guava that is cheaper and has better food safety regulations. Future research also needs to consider combining applications, i.e., composite and hurdle technology, to enhance shelf life and maintain postharvest characteristics such as consumer acceptance, commercial availability, cost-effectiveness, safety, and legal aspects of guava fruit, over the storage period. However, the implementation of some of these postharvest technologies should be expected in the future to minimize postharvest losses of guava and contribute to environmental sustainability.

## Figures and Tables

**Table 1:** Effect of MAP/CAP packaging on the shelf life of guava fruits.

Guava Variety	Packaging Technologies	Type of Package	Optimum Storage Condition	Storage Life	References
Guava cv. Pedro Santo	CAP	Plastic bucket	5 kPa O_2_ + 5 kPa CO_2_	28 days at 12.2 °C and 95% RH	[30]
Guava cv. Lucknow-49	CAP	50 µm LDPE	9% O_2_ + 5% CO_2_	42 days at 10 °C	[29]
Guava cv. Lucknow-49, *Allahabad safeda* and Apple Color	CAP	-	2.5, 5, 8, and 10 kPa O_2_ with 2.5, 5, and 10 kPa CO_2_ (balance N_2_)	30 days at 8 °C and 60–80% RH	[11]
Guava cv. Pedro Sato	CAP	Hermetic plastic containers	21, 15, 10, 5, and 1 kPa)	28 days at 12.5 °C	[85]
Guava cv. Baruipur	Active MAP	40 µm PVC	3 g ES and 46 g MS with a head space gaseous concentration of 12.6% O_2_ + 5% CO_2_	32 days at 4 °C	[91]
Guava cv. Baruipur	Passive MAP	45 µm BOPP film	5% O_2_ and 4% CO_2_	26 days at 10 °C	[24]
Guava	Active MAP	76.2 µm LDPE	Potassium permanganate embedded in silica crystals for modifying the gaseous concentration inside the package	49 days at 8 ± 2 °C	[92]
Guava cv. Allahabad Safeda	Passive MAP	45 µm PP with perforation	Perforations (five holes in each side of film with a size of 0.3 mm diameter) for modifying the gaseous composition	4 days at 25–28 °C and 60–70% R.H. and 28 days at 8–12 °C and 88–90% R.H.	[16]
Guava cv. Hisar Safeda	Passive MAP	50 µm LDPE	Films with selective permeability enhance the environment surrounding the guava	21 days at 7 ± 3 °C	[31]
Guava	Passive MAP	20 µm PP with perforation	12.5 mm perforation in films maintained the required gaseous conditions	20 days at 10 °C	[93]

**Table 3:** Essential-oil-based edible coatings for enhancing the shelf life of guava fruits.

Matrix	Best Combination	Effect of Coatings	Shelf Life	Deposition	References
Gum Arabic (GA) + Cinnamon Essential Oil (CEO) + Oleic Acid	10%GA + 1% OA + 1% CEO	Retention of fruit firmness; reduction in physiological loss of weight; lowering of browning index; enhancement of bioactive molecules such as phenolics and flavonoids and enhancement of antioxidant activity.	10 ± 1 °C and 90% relative humidity for 28 days	Dipping	[38]
Chitosan (C)+ Ruta graveolens Essential Oil (RGEO)	2% C + 1.5% RGEO	Microbial analysis shows a reduction of 2 log CFU/g in yeast and molds count; in situ growth inhibition of *Colletotrichum gloesporioides* by 70.71%; retention of fruit firmness; and reduction in physiological loss of weight.	12 days 24 ± 2 °C and relative humidity of 70%	Dipping	[142]
Gum Arabic (GA) + Cinnamon Essential Oil (CEO)	10% GA + 1% CEO	Reduction in loss of weight by 42.72% as compared to control sample. Retention of firmness, chlorophyll content, and caretonenoid content by 21.03%, 66.67%, and 56.7%, respectively, as compared to control sample; enhancement of ascorbic acid content (114.22 mg 100 g^−1^ FW).	28 days at (10 ± 1 °C, 90–95% RH followed by 1 day at room temperature	Dipping	[25]
Arabic Gum (AG) + Sodium Caseinate (SC) + Cinnamon Oil (CE) + Lemongrass Oil (LG)	5% AG + 1% SC + 2% CE + 2% LG	Decrease in enzyme activity of polyphenol oxidase (PPO) and peroxidase (POD); increase in antioxidant capacity; better retention of ascorbic acid and bioactive compounds such as phenolic and flavonoids.	35 days 4–7 °C and 80% RH	Dipping	[7]
Arabic Gum + Sodium Caseinate (SC) + Tulsi Extract (TE)	5%AG + 1% SC and 2.5% TE	The value of OTR (oxygen transmission rate) and WVTR (water vapor transmission rate) was lower than control sample, which resulted in delaying in ripening of fruit and extension of shelf life.	7 days at 28 ± 2 °C	Dipping	[17]
Groundnut Oil, Sesame Oil, Baobab Oil, Olive (*Olea europaea* L.) and Neem Oils	peanut and sesame oil coating	Extending the shelf life of guava fruits and controlling nutritional value, microbial growth, firmness, and appearance of the fruits.	Enhanced shelf life of guava fruits at Room conditions	Dipping	[143]
Cassava Starch (CS) + Chitosan (C) + *Lippia gracilis* Schauer Genotypes (EOM)	2.0% CS + 2.0% C and 1.0%, 2.0% or 3.0% EOM	Essential oil at all concentrations was effective in inhibiting Gram positive as well Gram negative bacteria; coating reduced the browning of guava; enhancement in L* value and reduction in a* and b* value during storage; and reduction in weight loss and better retention of firmness in coated sample	10 days at 25 °C 86–89% relative humidity	Dipping	[144]

L*, a* and b* represents the colour values of the tomato.

**Table 4:** Main outcomes of the effect of packaging technologies on guava fruits.

S.No.	Key Quality Attributes	Outcomes of the Effect of Packaging Technologies	References
1.	Weight Loss	Packaging technologies are more effective at controlling weight loss than unpackaged/uncoated fresh guava due to the water barrier properties offered by the packaging material that slows down the transpiration rate along with migration of water vapor from the surface of fresh produce to the external environment.	[11,24,33,147,149]
2.	Respiration Rate	Packaging technologies aim at modifying the gaseous atmosphere surrounding the guava in such a way that reduces the respiration rate and delays the onset of respiratory peak due to the selective permeability of O_2_ and CO_2_ offered by the packaging materials. The reduction in respiration rate delays the ripening-associated changes in the guava fruit during storage.	[17,21,33,34]
3.	Ethylene Biosynthesis	Packaging technologies such as MAP and edible coatings delay ethylene biosynthesis and its accumulation during the ripening of guava due to decrease in the respiration rate and reduction in the activity of various enzymes involved in biosynthesis of ethylene.	[34,129,155,156,157]
4.	Color	Retention of green color of the fresh guava in packaged form as compared to uncoated samples during the storage due to the lowering of respiration rate and inhibition of browning, causing enzymatic activity.	[24,25,144,158,160]
5.	Firmness	Packaging technologies are effective at retaining the firmness of fresh guava due to limiting the oxygen concentration, which in turn delays the solubilizing of pectin and slows down the activities of cell-walldegrading enzymes such as polygalacturonase (PG) and pectinesterase (PE).	[17,24,25,161,167,168,170]
6.	Total Soluble Solids (TSS) and Titrable Acidity (TA)	Packaging technologies slow downs the increase in TSS and TA during the storage of guava by lowering the respiration rate, which slows down the hydrolytic activity of enzymes associated with the hydrolysis of complex biomolecules such as carbohydrates and organic acids.	[7,24,38,129,162,163]
7.	Ascorbic Acid	Packaging technologies aim at delaying or preventing the oxidation of ascorbic acid by ascorbate oxidase due to limiting the oxygen concentration and improve the retention of ascorbic acid in fresh guava during storage.	[7,8,16,38]
8.	Microbial Decay	Packaging technologies offer an external barrier to fresh guava for the inhibition or reduction of microbial population. Edible coatings with antimicrobial agents are effective at reducing total yeast, mold, and bacterial counts and preventing microbial-spoilage-associated changes.	[5,136,138,171]
9.	Chill Injuries	Packaging technologies such as MAP and edible coatings have the potential to reduce the incidence of chilling injury in fresh guava by minimizing lipid peroxidation and browning-associated changes during low temperature storage.	[8,29,38,79,173,174]
10.	Sensory Properties	The application of packaging technologies on fresh guava fruit improves or maintains the overall sensory characteristics better than uncoated/unpackaged fruit due to the delay in ripening-associated changes induced by lowering of respiration rate and ethylene biosynthesis.	[8,37,177,178,179,180]

## Data Availability

Data is contained within the article.
